# Estrogenic Effects of the Extracts from the Chinese Yam (*Dioscorea opposite* Thunb.) and Its Effective Compounds in Vitro and in Vivo

**DOI:** 10.3390/molecules23020011

**Published:** 2018-01-23

**Authors:** Mengnan Zeng, Li Zhang, Miao Li, Beibei Zhang, Ning Zhou, Yingying Ke, Weisheng Feng, Xiaoke Zheng

**Affiliations:** 1 Department of Medicine, Henan University of Chinese Medicine, China; 17320138484@163.com (M.Z.); sonny.fairy.love@163.com (L.Z.); limiao1206sunny@163.com (M.L.); zhangs9426@163.com (B.Z.); zhoun0813@163.com (N.Z.); keyingying1988@163.com (Y.K.); 2 Collaborative Innovation Center for Respiratory Disease Diagnosis and Treatment & Chinese Medicine Development of Henan Province, China

**Keywords:** Chinese yam, adenosine, arbutin, estrogen-like effect

## Abstract

Background: The aim of this study was to explore the estrogenic effects of the extracts from Chinese yam and its effective compounds. Methods: The activity of the yam was investigated by the uterine weight gain of mice and a proliferation assay of breast cancer cell lines (MCF-7 cell); the estrogenic activity was comprehensively evaluated by a serum pharmacology experiment. The levels of estradiol (E2), follicle stimulating hormone (FSH), and luteinizing hormone (LH) were also measured. Western blot analysis and antagonist assays with faslodex (ICI182,780), methylpiperidino-pyrazole (MPP), Delta (9) –tetrahydrocannabinol (THC), and G-15 were used to explore the mechanism of the effects of the yam. To find the effective compounds of the yam which play a role in its estrogen-like effects, we used the same methods to study the effects of adenosine and arbutin. Results: The Chinese yam and two main compounds, adenosine and arbutin, have estrogen-like effects. The mechanism of the yam which plays a role in its estrogen-like effects was mainly mediated by the estrogen receptors ERα, ERβ, and GPR30; that of adenosine was mainly mediated by estrogen receptors ER*α* and ERβ, and that of arbutin was mainly mediated by estrogen receptors ERβ and GPR30. Conclusions: The Chinese yam has estrogen-like effects; adenosine and arbutin are two of the effective compounds in the yam which play a role in its estrogen-like effects.

## 1. Introduction

Ovarian function declines with age. As the estrogen level decreases, menstruation becomes disordered and amenorrhea follows. At this time, women gradually enter into menopause. Menopause usually occurs between the ages of 45 and 55 [1]. After women enter menopause, they may encounter a series of diseases and symptoms, such as menstrual disorder, mood swings, hot flashes, osteoporosis, Alzheimer’s disease, cardiovascular disease, etc. Long-term estrogen replacement therapy (ERT) plays a very important role in the treatment of perimenopausal symptoms. ERT relieves symptoms caused by estrogen decline and reduces the incidence of osteoporotic fracture and cerebral infarction [2]. However, long-term use of synthetic estrogen can cause endometrial cancer, breast cancer, and other diseases [3]; therefore, phytoestrogens have become a hotspot for research. Phytoestrogens such as isoflavones and isocoumarins exist in some plants and have estrogen-like effects. With structures similar to estrogen, phytoestrogens bind with estrogen receptors in vivo [4]. The aim of the current study was to investigate the pharmacological properties and the estrogen-like effects of a traditional Chinese pharmacopeial herb and to validate the potential beneficial effects of the herb.

*Dioscorea opposite* Thunb.—produced in Wenxian county, Jiaozuo city, Henan province, China—named Chinese yam (called yam in this paper), is a well-known edible and pharmaceutical food in China [5]. In addition, it has been widely used to promote human health and provide functional foods in traditional Chinese medicine (TCM). Modern pharmacological studies have shown that Chinese yam extracts have anti-inflammation and anti-oxidation properties, as well as the ability to lower blood pressure and blood sugar [6]. Extant literature supports the notion that the yam inhibits the mitogen-activated protein kinase (MAPK), protein kinase (Akt) and nuclear factor kappa-light-chain-enhancer of activated B cells (NFκB) signaling pathways [7], which are related to the development of disease. In TCM, medicine and food are used to promote human health and function in ways which can have estrogen-like effects. However, it is still to be clarified whether or not the Chinese yam has estrogen-like activity and which of its compounds are effective.

## 2. Results

### 2.1. The Influences of Chinese Yam on MCF-7 Cells

Compared with the control group, yam extract at the doses of 1, 10^−1^, 10^−2^, 10^−3^ mg/mL increased the proliferative events in the MCF-7 cell line. As a positive control, 17β-estradiol (17β-E2) also significantly increased the proliferative events in the MCF-7 cell line. The results are shown in Table 1.

### 2.2. The Influences of Chinese Yam on Immature Female Swiss Mice

Compared with the control group, the yam significantly increased the uterus coefficient. We also determined the levels of estradiol (E2), follicle stimulating hormone (FSH), and luteinizing hormone (LH) of the serum. The yam significantly increased the levels of E2 and FSH. As a positive control, estradiol valerate (EV) increased the uterus coefficient in mice, and the levels of E2, FSH and LH, significantly. The results are shown in Table 2.

### 2.3. Effect of Chinese Yam on the Expression of ERα, ERβ and GPR30 in the Uterus 

**Figure 1:**
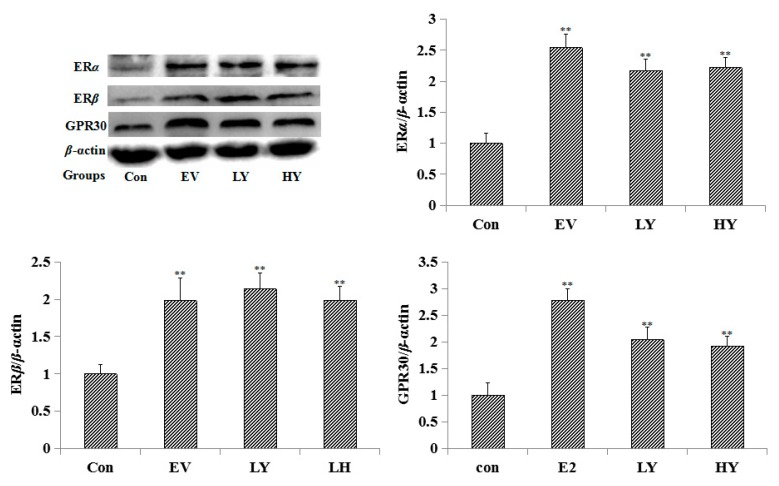
The expression of ERα, ERβ, and GPR30 in the uterus tested by the Western blot method (*n* = 3). LY: Low dose of yam, LH: High dose of yam. ** *p* < 0.01 compared to the control group.

The results of the Western blot showed that the low dose of yam (LY) (1630 mg/kg) and the high dose of yam (HY) (3260 mg/kg) significantly increased the expression of estrogen receptors ERα, Erβ, and GPR30 in the uterus. As a positive control, EV (0.33 mg/kg) also increased the expression of ERα, ERβ, and GPR30 in the uterus. The results are shown in Figure 1.

### 2.4. Effect of ICI182,780, MPP, THC and G15 on the Proliferation of Yam-Stimulated MCF-7 Cells 

**Figure 2:**
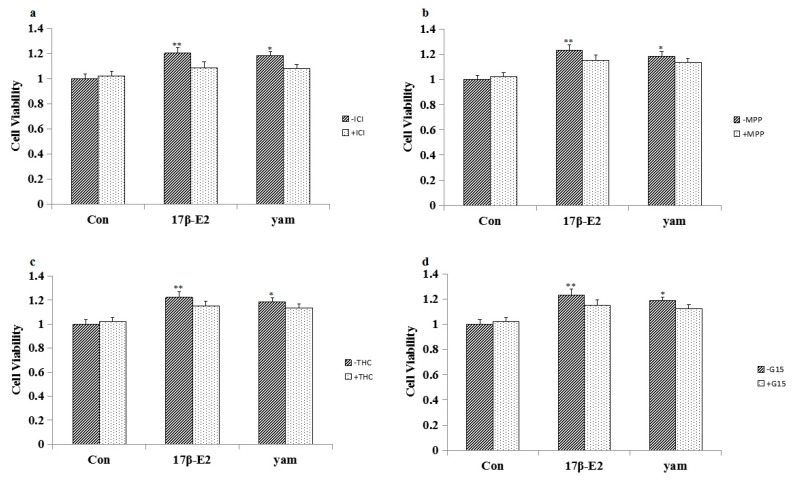
Blockade of ER and GPR30 completely abolished the proliferation of yam-stimulated MCF-7 cells (*n* = 4). * *p* < 0.05; ** *p* < 0.01 compared to controls. (**a**) Effects of ICI182,780 on the proliferation of yam-stimulated MCF-7 cells. (**b**) Effects of MPP on the proliferation of yam-stimulated MCF-7 cells. (**c**) Effects of THC on the proliferation of yam-stimulated MCF-7 cells. (**d**) Effects of G-15 on the proliferation of yam-stimulated MCF-7 cells.

To confirm whether MCF-7 cell proliferation induced by the yam was mediated by ER and GPR30, MCF-7 cells were exposed to yam extract (10^−2^ mg/mL) with the unspecific ER antagonist ICI 182780 (1 μM), specific ERα antagonist MPP (1 μM), specific ERβ antagonist THC (1 μM), or specific GPR30 antagonist G-15 (1 μM). Results showed that the blockade of ER and GPR30 completely abolished the proliferation of yam-stimulated MCF-7 cells, suggesting that the proliferation of yam-stimulated MCF-7 cells was mediated by ERα, ERβ and GPR30. The results are shown in Figure 2.

### 2.5. The Influences of Adenosine and Arbutin on MCF-7 Cells

Compared with the control group, adenosine and arbutin increased the proliferative events in the MCF-7 cell line. As a positive control, 17β-E2 and the yam increased the proliferative events in the MCF-7 cell line significantly. These results are shown in Table 3.

### 2.6. The Influences of Adenosine and Arbutin on Immature Female Swiss Mice

Compared with the control group, adenosine and arbutin significantly increased the uterus coefficient (36.21% and 27.86%, respectively). We also determined the levels of E2, FSH, and LH of serum. Adenosine and arbutin significantly increased the levels of E2 and FSH. As a positive control, EV and the yam increased the uterus coefficient in mice (126.76% and 33.09%, respectively), and the E2 (38.24% and 31.60%, respectively) and LH (24.22% and 37.75%, respectively) levels in serum significantly. The results are shown in Table 4.

### 2.7. Effect on the Expression of ERα, ERβ and GPR30 in MCF-7 Cells and the Uterus

**Figure 3:**
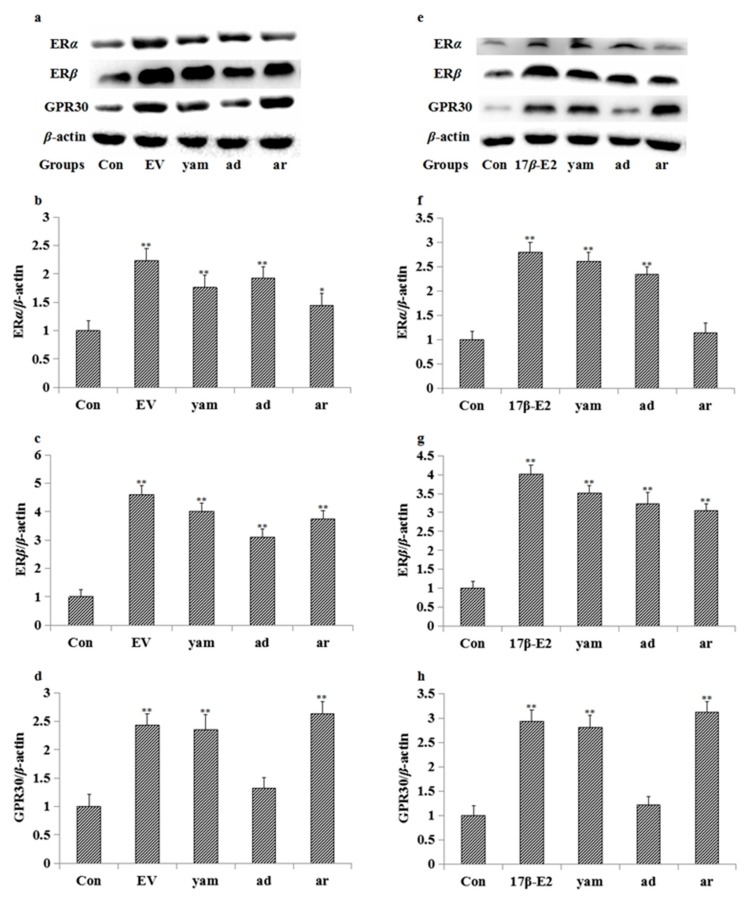
The expression of ERα, ERβ, and GPR30 in the uterus and MCF-7 cells tested by the Western blot method (*n* = 3). ad: adenosine, ar: arbutin. (**a**–**d**) were the expression of ERα, ERβ, and GPR30 in the uterus. (**e**–**h**) were the expression of ERα, ERβ, and GPR30 in MCF-7 cells. * *p* < 0.05; ** *p* < 0.01 compared to the control group.

The results of the Western blot showed that adenosine significantly increased the expression of ERα and ERβ in the uterus and MCF-7 cells, and arbutin significantly increased the expression of ERβ and GPR30 in the uterus and MCF-7. As a positive control, EV (or 17β-E2) and the yam increased the expression of ERα, ERβ and GPR30 in the uterus and MCF-7 cells. The results are shown in Figure 3.

### 2.8. Effect of ICI182,780, MPP, THC and G-15 on Adenosine- and Arbutin-Stimulated MCF-7 Cell Proliferation and the Expression of ERα, ERβ and GPR30 in MCF-7 Cells

**Figure 4:**
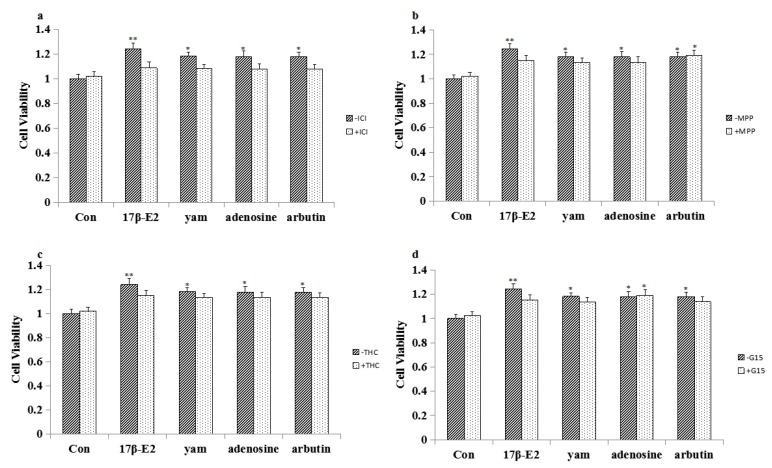
Effect of ICI 182780, MPP, THC and G-15 on MCF-7 cell proliferation (*n* = 3). (**a**) Effects of ICI182,780 on the proliferation of adenosine- and arbutin-stimulated MCF-7 cells. (**b**) Effects of MPP on the proliferation of adenosine- and arbutin-stimulated MCF-7 cells. (**c**) Effects of THC on the proliferation of adenosine- and arbutin-stimulated MCF-7 cells. (**d**) Effects of G-15 on the proliferation of adenosine- and arbutin-stimulated MCF-7 cells. * *p* < 0.05; ** *p* < 0.01 compared to the control group.

**Figure 5:**
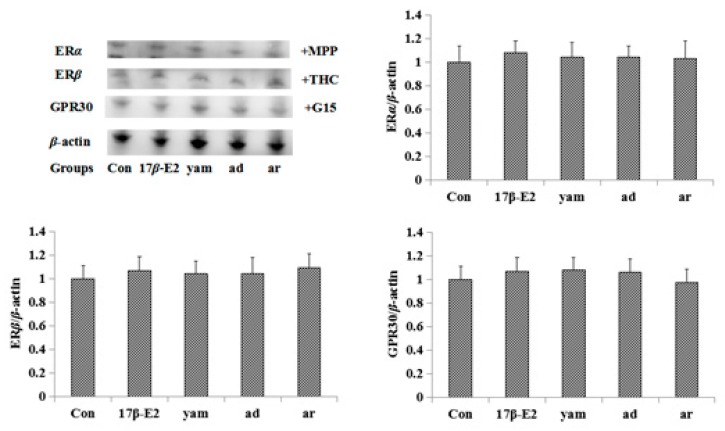
Effect of MPP, THC and G-15 on the expression of ERα, ERβ and GPR30 in MCF-7 cells (*n* = 3). The MPP, THC and GPR30 were added 30 minutes before treatment of 17β-E2, yam, adenosine and arbutin.

To test whether the MCF-7 cell proliferation induced by adenosine and arbutin were mediated through ER and GPR30, MCF-7 cells were exposed to the unspecific ER antagonist ICI182,780 (1 μM), the specific ERα antagonist MPP (1 μM), the specific ERβ antagonist THC (1 μM), and the specific GPR30 antagonist G-15 (1 μM). Blockage of ER completely abolished the proliferation of MCF-7 cells, suggesting that ERα and ERβ mediate the proliferative effects of adenosine. Blockage of ERβ and GPR30 completely abolished the proliferation of MCF-7 cells, suggesting that ERβ and GPR30 mediate the proliferation effects of arbutin in MCF-7 cells. These results are shown in Figure 4. The expression of ERα, ERβ, and GPR30 in MCF-7 cells induced by adenosine and arbutin with the antagonists MPP, THC or G-15 is shown in Figure 5.

### 2.9. Pharmacological Experiment with Serum

The results of the pharmacological experiment showed that the serum of mice treated with the yam, adenosine, and arbutin significantly promoted the proliferation of MCF-7 cells (25.6%, 25.2% and 23.2% respectively). The results are shown in Table 5.

### 2.10. Quantitative Analysis of Adenosine and Arbutin

**Figure 6:**
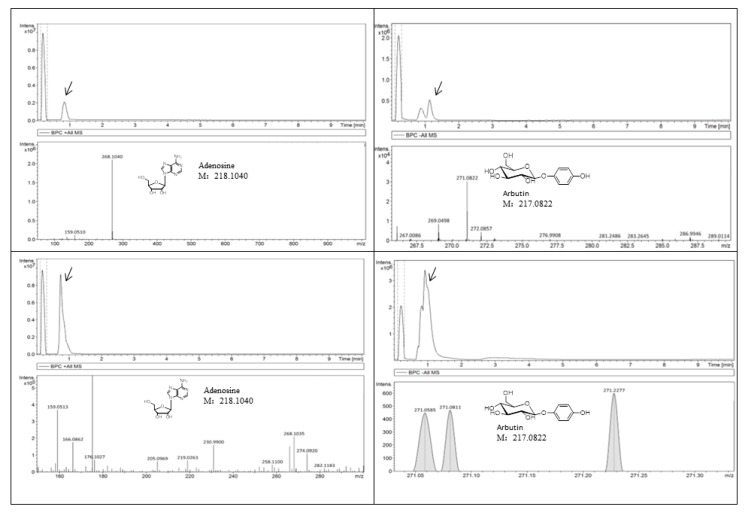
HPLC-MS fingerprint of the yam, adenosine and arbutin. Adenosine and arbutin were verified as genuine resveratrol tetramers in the Chinese yam.

Adenosine and arbutin were verified as genuine resveratrol tetramers since they were detectable by assays of liquid chromatography-mass spectrometry (LC-MS) (Figure 6) in the yam. The adenosine and arbutin content in the extracts from the Chinese yam was 0.15‰ and 0.08‰ respectively.

## 3. Discussion

Perimenopausal syndrome is a common disease in women before and after menopause, which seriously affects their physical and mental health and daily life. Estrogen replacement therapy (ERT) can alleviate the symptoms of perimenopause syndrome, but ERT can lead to increased risk of breast cancer, endometrial cancer, and cardiovascular disease. Phytoestrogens are those that can bind to and activate estrogen receptors, having estrogen-like or antiestrogenic activity. They are known as selective estrogen receptor modulators (SERMs) because of their targeted role. According to their characteristics, phytoestrogens are expected to be potential drugs for the treatment of perimenopausal syndrome. An increasing number of researchers are investigating the relationship between phytoestrogen intake and perimenopausal syndrome [8,9,10]. In fact, estrogens are already being used in the treatment of various diseases such as osteoporosis, hyperlipidemia [11], cardiovascular disease [12], and Alzheimer’s disease (AD) [13]. Traditional Chinese medicine, which is rich in phytoestrogens, is more concentrated on tonic medicine and blood medicine, and yams have been widely used in TCM to promote human health and provide a functional food in China. In this study, we evaluated the estrogen-like effects of the Chinese yam and found the effective compounds which cause these. 

Earlier methods of studying ovarian hormones were based on the growth of the uteri in normal or castrated immature rabbits [14,15] until Astwood [16,17] determined that the uterine weight response to gonadotropins in immature albino mice is much more uniform and more sensitive. Uterus growth tests in mice were established to detect the activity of estrogen. In this study, the ratio of wet weight to body weight (uterus coefficient) of the uterus of immature female mice was used as an index to evaluate estrogen-like activity. The results showed that the yam (1630 mg/kg and 3260 mg/kg), adenosine (50 mg/kg), and arbutin (50 mg/kg) significantly increased the uterus coefficient and the levels of E2 and FSH, suggesting that the yam, adenosine, and arbutin had an estrogen-like effect in vivo.

The serum pharmacological method is generally used in herb studies. Mouse serum pharmacological experiments avoid interference from the physical and chemical properties of a drug and produce a pharmacological effect of the real process [18]. The yam, adenosine, and arbutin significantly promoted the proliferation of MCF-7 cells and showed an estrogen-like effect, which was consistent with the effect of the yam, adenosine, and arbutin on the uterine weight gain of mice.

Both the physiological and pathological effects of estrogen are mediated by estrogen receptors, which are divided into two types: the genomic nuclear estrogen receptors α (ERα) and β (ERβ), as well as the non-genomic estrogen receptor (GPR30) and possibly other non-genomic receptors. The expression of the estrogen receptor protein in the uterus was studied by the Western blot method to explore the way in which the molecular mechanism of this drug exerts an estrogen-like effect. The results showed that EV (0.33 mg/kg) and the yam (1630 mg/kg, 3260 mg/kg) significantly increased the expression of ERα, ERβ, and GPR30 protein in the uterus, suggesting that the yam had an estrogen-like effect in vivo through ERα, ERβ, and GPR30. Adenosine (50 mg/kg) significantly increased the expression of ERα and ERβ protein in the uterus, and arbutin (50 mg/kg) increased the expression of ERβ and GPR30 protein in the uterus, suggesting that adenosine had an estrogen-like effect in vivo through ERα and ERβ, and arbutin had an estrogen-like effect in vivo through ERβ and GPR30.

MCF-7 cells are estrogen receptor-positive human breast cancer cell lines that are specifically stimulated by estrogen or estrogen-like substances and are the cell line most commonly used to detect estrogen-like activity [19]. In this study, different doses of yam (1, 10^−1^, 10^−2^, 10^−3^ mg/mL) increased the proliferative events in the MCF-7 cell line, and adenosine (5 μM) and arbutin (5 μM) also increased the proliferative events in the MCF-7 cell line. In another set of experiments, ICI182,780 [20], MPP [21], THC [22], and G-15 [23] were used to evaluate whether the observed effects elicited by the yam, adenosine and arbutin were mediated by ER and GPR30. The results showed that blockage of ER and GPR30 can abolish the proliferation of MCF-7 cells, suggesting that ERα, ERβ and GPR30 have roles in mediating the proliferation effects of the yam in MCF-7 cells. Blockage of ER can abolish the proliferation of MCF-7 cells, suggesting that ERα and ERβ have roles in mediating the proliferation effects of adenosine in MCF-7 cells. Blockage of ERβ and GPR30 can abolish the proliferation of MCF-7 cells, suggesting that ERβ and GPR30 have roles in mediating the proliferation effects of arbutin in MCF-7 cells. 

Additionally, the results of the Western blot showed that 17β-E2 (1 μM) and the yam (10^−2^ mg/mL) significantly increased the expression of ERα, ERβ and GPR30 protein in MCF-7 cells, while adenosine significantly increased the expression of ERα and ERβ, and arbutin significantly increased the expression of ERβ and GPR30 in MCF-7 cells. Results were consistent with the effects of the yam, adenosine and arbutin on the uterus of mice. All results suggest that adenosine exerts an estrogen-like effect through the estrogen receptors ERα and ERβ, and arbutin exerts an estrogen-like effect through the estrogen receptors ERβ and GPR30. However, the yam exerts estrogen-like effects through the estrogen receptors ERα, ERβ and GPR30. 

In this study, the results showed that the EV significantly increased the uterus coefficient of mice; the uterus of the EV group also exhibited edema and endometrial thickening. We speculated that the yam, adenosine, and arbutin did not have the side effects of EV. In this study, the uterus of the EV group appeared to have edema and endometrial thickening, which could be caused by the promotion of vascular endothelial growth factor (VEGF) by estradiol valerate in the uterus. Additionally, estrogen can enhance the permeability of blood vessels, induce angiogenesis and endothelial cell growth, and inhibit apoptosis [24].

Before now, no one proved the estrogenic effects of adenosine and/or arbutin. Adenosine is a ubiquitous purine ribonucleoside with important regulatory activities, mainly cytoprotective functions. In the brain, adenosine is an endogenous distress signal that modulates many neuronal and pathophysiological conditions [25,26]. Arbutin—which is mainly extracted from leaves of the Bearberry and is also found in some fruits and other plants— is a hydroquinone glycoside, which has been reported to have signaling abilities such as reducing the melanin content of mouse B16 melanoma cells at concentrations in the range of 0.014.1 mM [27]. Several in vitro and in vivo studies revealed the anti-melanogenic activity of arbutin, which can be useful in hyperpigmentation therapy [28]. In our preliminary laboratory study, adenosine and arbutin were isolated from the yam. In the present study, we assessed the estrogen-like effects of the yam, and found that the effective compounds were adenosine and arbutin. 

In total, we isolated adenosine and arbutin, which were the main compounds of the yam; the uterine growth test in mice and the in vitro MCF-7 cell proliferation experiments together prove that adenosine and arbutin have an estrogen-like effect. Adenosine and arbutin were the effective compounds of the yam that exerted an estrogen-like effect. The results of the ICI182,780, MPP, THC and G-15 antagonist experiments and the Western blot analysis confirm that the estrogen-like effect of the yam is mainly mediated by the estrogen receptors ERα, ERβ and GPR30; that of adenosine is mainly mediated by estrogen receptor ERα and ERβ, and that of arbutin is mainly mediated by estrogen receptor ERβ and GPR30. 

Overall, Chinese yam has estrogen-like effects and the effective compounds were adenosine and arbutin. Chinese yam, adenosine, and arbutin do not have the side effects of estradiol valerate, and may be developed into a new drug for the treatment of perimenopausal syndrome.

## 4. Materials and Methods 

### 4.1. Plant Material and Reagents

Tuberous roots of the Chinese yam were collected from Jiaozuo city, China, and identified by Professor Chen Suiqing, Henan University of Traditional Chinese Medicine. A voucher specimen (No. 20160715A) was deposited in the Research Department of Natural Medicinal Chemistry, School of Pharmacy, Henan University of Traditional Chinese Medicine. Dry tuberous roots of the Chinese yam (1.0 kg) underwent extraction three times with H_2_O (10 L × 3, 1.5 h each time) at 100 °C. Evaporation of the solvent under reduced pressure provided aqueous extracts (0.163 kg). Compounds such as allantoin, arbutin, adenosine, and niacinamide were separated from extracts from Chinese yam in our previous studies; the arbutin and adenosine content was 1.5‰ and 0.8‰ respectively.

The reagents were Dulbecco’s modified eagle medium (DMEM, Gibco, Pittsburgh, PA, USA); heat-inactivated fetal calf serum(HyClone, Logan, UT, USA); 17β-E2 (positive drugs for cell experimentation, Sigma, Louis, MO, USA); estradiol valerate (EV, positive drugs for cell experimentation, Bayer medical, Shanghai, China); methyl thiazolyl tetrazolium (MTT) and dimethyl sulfoxide (DMSO) (Amresco, Seattle, WA, USA); Estradiol (E2), luteinizing hormone (LH), Follicle stimulating hormone (FSH) (R＆D, Minneapolis, MN, USA); ICI182,780, MPP, THC and G-15 (Tocris, Bristol, UK). A microplate reader(Bio-Rad, Hercules, CA, CA, USA) was also used.

### 4.2. Cell Culture and Treatment

The MCF-7 was grown in Dulbecco’s modified Eagle’s medium supplemented with 2 mM l-glutamine, 50 units/mL penicillin, 50 μg/mL streptomycin, and 10% heat-inactivated fetal calf serum. Prior to experiments involving treatment with estrogens, cells were cultured in a hormone-free medium (PAnol red-free Dulbecco’s modified Eagle’s medium) with 10% (*v*/*v*) charcoal-stripped fetal bovine serum for 3 days at 37 °C in a water-saturated 5.0% CO_2_ incubator(Thermo Scientific, Waltham, MA, USA). MCF-7 cells were seeded in 96-well plates in PAnol red-free Dulbecco’s modified Eagle’s medium with 10% (*v*/*v*) charcoal-stripped fetal bovine serum. The density of the cells in each plate was 3 × 10^4^ cells/mL. Then, the cells were divided into the control group, the 17β-E2 group (1 μM), the different doses of yam, and the adenosine and arbutin groups. Twenty-four hours later, the cell vitality was detected by MTT. MCF-7 cells were seeded in 96-well plates as previously described. Cells were exposed for 24 h to 17β-E2, yam, adenosine and arbutin. In another experiment, the ER-unspecific antagonist faslodex (ICI182,780, 1 μM), the specific ERα antagonist methylpiperidino-pyrazole (MPP, 1 μM), the specific ERβ antagonist Delta (9) -tetrahydrocannabinol (THC, 1 μM), or the specific GPR30 antagonist (G-15, 1 μM) were added 30 min before the treatments of 17β-E2, yam, adenosine and arbutin to evaluate whether the observed effects elicited by the yam, adenosine and arbutin were mediated by ER and GPR30. Twenty-four hours later, the cell vitality was detected by MTT.

### 4.3. MTT Assay

An amount of 20 μL of MTT solution (12.08 × 10^−3^ mmol/L) was added to each well. Four hours later, the medium was removed and 150 μL of DMSO was added. The cells were then shocked for 600 s, using a microplate reader(Bio-Rad, Hercules, CA, USA) at a wavelength of 490 nm to measure the absorbance.

### 4.4. Animals

The study was conducted in accordance with the Experimental Animal Administration regulations issued by the State Committee of Science and Technology of the People’s Republic of China. The ethical approval reference number of the study is SYXK2010-004. Immature female Swiss mice (9–11 g) were obtained from Beijing Vital River Laboratory Animal Technology Co., Ltd, and were housed under standard environmental conditions. Forty immature female Swiss mice were divided into 4 groups; each group included 10 mice. The 4 groups were as follows: the control group, the EV group (0.33 mg/kg), a group with a low dose of yam (LY, 1630 mg/kg), and a group with a high dose of yam (HY, 3260 mg/kg). The dosing volume for each mouse was 0.1 mL/10 g. The doses were administered as described above for 7 days continuously. Eighteen hours after the last administration, the animals were anesthetized with ketamine hydrochloride, and heart punctures were performed to collect blood with 1 mL syringes. The carefully dissected uteri were weighed on a sensitive torsion balance. The mouse uterine weight gain test for adenosine and arbutin was the same test as for the yam.

### 4.5. Western Blot

For Western blot analysis, MCF-7 cells were seeded in a 6-well plate (5 × 10^5^) as previously described. Cells were lysed in radioactive immunoprecipitation assay buffer. Proteins from the uteri were extracted with a mammalian protein extraction kit (Beijing ComWin Biotech Co., Ltd., Beijing, China). Proteins were quantified using the Bradford protein assay kit (Wuhan Boster Biological Technology.,Ltd., Wuhan, China) The Western blot method was applied to detect the expression levels of ERα, ERβ and GPR30. The protein samples were separated by SDS-PAGE. An amount of 40 mg of each protein sample was loaded into the gel and transferred to a poiy vinylidene fluoride (PVDF) membrane. Then, non-fat milk was used to block the membrane for 2 h at room temperature; afterwards, the membrane was incubated with a primary antibody (ERα 1:500; ERβ 1:500; and GPR30 1:500) overnight at 4 °C. After being washed in Tris Buffered Saline with Tween-20 (TBST) buffer, the membrane was incubated with a secondary antibody for 1 h at room temperature. The intensity of the proteins was quantified using Gene Tools.

### 4.6. ELISA

For the ELISA assay, a 100 mm × 20 mm Petri dish was used. Serum was collected and used to detect the levels of E2, FSH, and LH according to the respective manufacturer’s instructions. 

### 4.7. Serum Pharmacology Experiment

The mouse serum for each group was prepared as follows: the blood was stored at 4 °C overnight, after centrifugation at 2000 r/min for 10 min and careful isolation of the serum. The serum was inactivated at 56 °C for 30 min, filtered through a 0.22 μm microfiltration membrane filter, and stored at −20 °C. The experiments on MCF-7 cell proliferation were performed as described above, but the MCF-7 cells were seeded in 96-well plates in phenol red-free Dulbecco’s modified Eagle’s medium with 10% (*v*/*v*) mouse serum. The viability of each group was compared after 24 h.

### 4.8. Quantitative Analysis of Adenosine and Arbutin

The LC system comprised of an Acclaim^TM^ RSLC 120 C_18_ column (2.2 µm, 2.1 × 100 mm; Thermo Scientific, Waltham, MA, USA). The mobile phase was composed of solvent A (0.1% formic acid–water) and solvent B (acetonitrile) with a gradient elution (0–3 min, 95–85% A; 3–6 min, 85–70% A; 6–8 min, 70–10% A; 8–10 min, 10–95% A). The flow rate of the mobile phase was 0.3 mL/min. The column temperature was maintained at 40 °C, and the sample manager temperature was set at 4 °C. Mass spectrometry was performed on a Quadrupole Time-of-Flight Mass Spectrometer (Q-TOF-MS; maXis HD, Bruker, Karlsruhe, Germany) using an ESI source. The scanning mass range (*m/z*) was from 50 to 1500 with a spectra rate of 1.00 Hz. The capillary voltage was set at 3500 V and 3200 V for positive and negative modes, respectively. The pressure of the nebulizer was set at 2.0 Bar, the dry gas temperature at 230 °C, and the continuous dry gas flow rate at 8 L/min.

### 4.9. Statistical Analysis

Analyses were performed using SPSS 18.0 (IBM, New York, NY, USA). Statistical significance was assessed in comparison with the respective control for each experiment using one-way ANOVA. *p*-values of less than 0.05 were accepted as significant.

## 5. Conclusions

The Chinese yam has estrogen-like effects; adenosine and arbutin are two of the effective compounds in the yam which play a role in its estrogen-like effects.

## Figures and Tables

**Table 1:** Effect of yam extract on MCF-7 cell proliferation (x ± SD, *n* = 4).

Groups	Dose	Cell Viability
Con	—	1.00 ± 0.021
17β-E2(μM)	1	1.25 ± 0.015 **
Yam (mg/mL)	1	1.31 ± 0.023 **
	10^−1^	1.26 ± 0.028 **
	10^−2^	1.19 ± 0.022 *
	10^−3^	1.17 ± 0.030 *
	10^−4^	1.09 ± 0.027

* *p* < 0.05; ** *p* < 0.01 compared to the control group.

**Table 2:** Effect on uterus coefficient, estradiol (E2), follicle stimulating hormone (FSH), and luteinizing hormone (LH) (x ± SD, *n* = 10).

Groups	Dose (mg/kg)	Uterus Coefficient (%)	E2 (pmol/L)	FSH (mIU/mL)	LH (mIU/mL)
Con	—	0.1002 ± 0.009	31.12 ± 3.01	45.32 ± 5.90	3.69 ± 0.69
EV	0.33	0.2515 ± 0.021 **	38.94 ± 2.98 **	53.17 ± 4.97 **	4.77 ± 0.74 **
LY	1630	0.1227 ± 0.027 *	39.21 ± 3.97 **	55.13 ± 4.26 **	3.54 ± 0.29
HY	3260	0.1269 ± 0.023 *	42.09 ± 5.04 **	56.13 ± 3.26 **	3.61 ± 0.48

EV: Estradiol valerate, positive control; LY: Low dose of yam; HY: High dose of yam. * *p* < 0.05; ** *p* < 0.01 compared to the control group.

**Table 3:** The influence on MCF-7 cell proliferation (x ± SD, *n* = 4).

Groups	Dose (μM)	Cell Viability
Con	—	1.00 ± 0.021
17β-E2	1	1.27 ± 0.016 **
Yam (mg/ml)	10^−2^	1.21 ± 0.024 *
adenosine	5	1.20 ± 0.028 *
	10	1.25 ± 0.022 **
arbutin	5	1.19 ± 0.030 *
	10	1.25 ± 0.027 **

* *p* < 0.05; ** *p* < 0.01 compared to the control group.

**Table 4:** Effect on uterus coefficient, E2, FSH and LH (x ± SD, *n* = 10).

Groups	Dose (mg/kg)	Uterus Coefficient (%)	E2 (p mol/L)	FSH (mIU/mL)	LH (mIU/mL)
Con	—	0.0994 ± 0.013	29.05 ± 5.11	45.32 ± 5.90	4.55 ± 0.54
EV	0.33	0.2254 ± 0.015 **	40.16 ± 8.09 **	56.30 ± 5.12 **	5.14 ± 0.58 **
yam	1630	0.1323 ± 0.036 *	38.23 ± 1.67 **	62.43 ± 7.72 **	4.16 ± 0.42
adenosine	50	0.1354 ± 0.023 *	42.96 ± 5.36 **	60.14 ± 6.17 **	4.07 ± 0.41
arbutin	50	0.1271 ± 0.018 *	38.39 ± 3.81 **	58.26 ± 6.32 **	4.19 ± 0.39

* *p* < 0.05; ** *p* < 0.01 compared to the control group.

**Table 5:** Effect of mouse serum on the proliferation of MCF-7 cells (x ± SD, *n* = 4).

Groups	Cell Viability
Con	1.00 ± 0.021
E2	1.23 ± 0.022 **
Yam	1.25 ± 0.019 **
Adenosine	1.24 ± 0.027 **
Arbutin	1.21 ± 0.030 **

* *p* < 0.05; ** *p* < 0.01 compared to the control group.

## Abbreviations

MCF-7 cell	Breast adenocarcinoma cell line
LH	Luteinizing hormone
FSH	Follicle stimulating hormone
ICI182,780	Faslodex
MPP	Specific ERα antagonist methylpiperidino-pyrazole
THC	Specific ERβ antagonist Delta (9) –tetrahydrocannabinol
G-15	Specific GPR30 antagonist
TBST	Tris Buffered Saline with Tween-20
MTT	Methyl thiazolyl tetrazolium
PBS	Phosphate Buffer Saline
DMSO	Dimethyl sulfoxide
LC-MS	Liquid chromatography-mass spectrometry
ERT	Estrogen replacement therapy
TCM	Traditional Chinese Medicine
17β-E2	17-beta-estradiol
PVDF	Poiy vinylidene fluoride
AD	Alzheimer’s disease
VEGF	vascular endothelial growth factor
SERMs	Selective estrogen receptor modulators
